# Mucormycosis in children with cancer and hematopoietic cell transplant—A single center cohort study

**DOI:** 10.1371/journal.pone.0297590

**Published:** 2024-02-09

**Authors:** Gabriela A. Marón, Kengo Inagaki, Alicia Rodriguez, Katherine M. Knapp, Randall T. Hayden, Elisabeth E. Adderson

**Affiliations:** 1 Department of Infectious Diseases, St. Jude Children’s Research Hospital, Memphis, Tennessee, United States of America; 2 Department of Pediatrics, University of Tennessee Health Sciences Center, Memphis, Tennessee, United States of America; 3 Department of Pathology, St. Jude Children’s Research Hospital, Memphis, Tennessee, United States of America; Sathyabama Institute of Science and Technology, INDIA

## Abstract

Although mucormycosis is an important cause of morbidity and mortality in children with cancer, our understanding of the typical characteristics of these infections is incomplete. We reviewed all cases of mucormycosis diagnosed at a single pediatric cancer center over 5 decades to identify the clinical features of mucormycosis in pediatric oncology patients and to identify risk factors for mortality. There were 44 cases of mucormycosis diagnosed between 1970–2019. Most patients (89%) had hematological malignancies and a history of prolonged and severe neutropenia (91%). In this series, hyperglycemia and exposure to corticosteroids were common. Pulmonary (36%) and disseminated infections (32%) were most common; rhino-orbital-cerebral infections were relatively infrequent (11%). *Rhizopus* spp. was the most common etiological agent (40%) followed by *Mucor* spp. (31%), and *Cunninghamella* spp. (19%). Overall mortality was 44% and 51% and attributable mortality was 39% and 41% at the end of antifungal therapy and end of follow up, respectively. Attributable mortality fell to 18% in 2010–2019, from 58–60% in previous decades; adjunctive surgery was associated with decreased mortality. Mortality remains unacceptably high despite aggressive antifungal therapy and adjunctive surgery, suggesting novel therapeutic strategies are needed.

## Introduction

Mucormycosis, previously also termed zygomycosis, is an acute or subacute invasive fungal infection caused by members of the order Mucorales [1, 2]. Over the past 5 decades, rates of mucormycosis have increased, while overall mortality rates (36–54%) of these infections have remained stable [3–6]. Reports suggest 7–16% of cases of mucormycosis occur in children, and 16–52% of pediatric mucormycosis is estimated to occur in children with cancer [4–9]. While the general characteristics of mucormycosis in children have been described, there have been only a small number of studies describing these infections in children with cancer, a population at high risk, and some of these have focused exclusively on children with hematological malignancies [6, 8, 10–15]. Since an appreciation of any distinctive characteristics of infection in this population may improve management, we reviewed all cases of mucormycosis in children with cancer or hematopoietic stem cell transplant (HCT) diagnosed over a 50-year period at a single cancer center.

## Methods

Patients with mucormycosis were identified among patients with malignant diseases or those who had received hematopoietic stem cell transplant (HCT) by searching health information records, diagnostic microbiology laboratory logs, and departmental databases. Only proven infections, as defined by the Invasive Fungal Infections Group from the European Organization for Research and Treatment of Cancer and the Mycoses Study Group and Education Consortium (EORTC/MSG) were included (i.e., histologically by the presence of hyphae or melanized yeast-like forms accompanied by evidence of associated tissue damage, by culture of sterile media or blood, or by amplification and sequencing of fungal DNA by PCR when molds are seen in formalin-fixed paraffin-embedded tissue) [16]. Cultures of tissues and body fluids were processed and incubated according to standard diagnostic microbiological methods of the era. Fungi were identified by their macroscopic and microscopic appearance, biochemical reactions, and or DNA amplification and sequencing either at the hospital’s or reference laboratories. Demographic and clinical characteristics were abstracted from patients’ health information records. Data was accessed for research purposes on multiple occasions from 11/2013 to 8/2022. Authors had access to information that could identify individual patients during data collection. The St. Jude Children’s Research Hospital Institutional Review Board approved this study with a waiver of consent. Rhino-orbital-cerebral involvement was categorized, when possible, as previously described [6, 16]. Disseminated infections were defined as those involving 2 or more noncontiguous sites; no attempt was made to assign a primary site of infection. Neutropenia was defined as an absolute neutrophil count of <1,000 per mm^3^ for any duration of time in the 30 days prior to the onset of infection. Hyperglycemia was defined as any elevation in blood glucose above the normal age-related value and corticosteroid and antifungal use as any duration of use in the 30 days prior to the onset of infection. Responses to antifungal therapy at 6 weeks, 12 weeks, and at the end of antifungal therapy and follow up were defined according to consensus criteria [17]. In the absence of a gold standard for attributable mortality, experienced clinicians reviewed patients’ clinical notes, and laboratory and pathologic results as available at the time of death. Investigators independently assessed the contribution of infection to mortality; differences were resolved by consensus of a subgroup of investigators (G.A.M, K.I., E.E.A.). Death was attributed to infection when no other contributing factors were present (e.g., pulmonary hemorrhage in the setting of a stable malignancy). Mucormycosis was considered to have contributed to death if other significant medical co-morbidities were present at the time of death (e.g., disseminated infection in the setting of severe graft vs. host disease post-hematopoietic stem cell transplant). Mucormycosis-associated deaths included those attributed or contributed to by mucormycosis.

Fungal nomenclature was updated, when feasible, to reflect changes to the International Code of Nomenclature adopted by the 18^th^ International Botanical Congress [18].

### Statistical analysis

Demographic and clinical characteristics were summarized by descriptive statistics and compared using the Wilcoxon rank sum or Kruskal-Wallis test for continuous variables and the Chi-square or Fisher’s exact test for categorical variables. Infection rates are expressed as numbers of infections per numbers of patients with cancer cared for at St. Jude in that calendar year. All available data were included in the analysis. Penalized logistic regression was used to examine the individual effects of demographic and treatment characteristics on attributable mortality, including variables with p<0.05 in univariate analyses [19]. Analysis was performed using Stata V16.1 (StataCorp, College Station, TX). All statistical tests were two-sided; P<0.05 was considered statistically significant.

## Results

A total of 44 EORTC/MSG-defined proven infections were identified among 43 patients S1 Table. During the same period 3 patients met EORTC/MSG criteria for probable infections and were not included in the analysis. One patient (episodes 15 and 16) developed disseminated *Mucor* spp. infection, including rhino-orbital-cerebral (ROC) and pulmonary involvement, during induction therapy for acute lymphoblastic leukemia (ALL). Complete radiological and clinical resolution of her infection was achieved. A year later her leukemia relapsed; induction therapy was again complicated by histologically confirmed, but culture-negative, ROC mucormycosis. It is uncertain whether the second episode represented a new or recurrent infection; it was considered to be a distinct episode in the analysis.

The number of infections increased from 4.6 per 1000 persons with cancer in the 1970’s to 10.9 per 1000 persons with cancer in the 2000’s and 10.0 between 2010–2019. Most infections (n = 31, 70%) were confirmed by positive cultures. A total of 23 patients (52%) were male Table 1. The most common underlying disorder was a hematological malignancy (n = 39, 89%), most commonly ALL (n = 19, 43%); other patients had solid or central nervous system tumors (n = 3) or had undergone HCT for treatment of a non-malignant disorder (n = 2). Among patients with hematological malignancies, 9 were relapsed and 14 were receiving induction therapy. Eleven patients had undergone HCT; 6 of 9 recipients of allogeneic transplants had active graft versus host disease at the time of infection. Neutropenia, at the time of diagnosis (n = 39, 91%) and in the 30 days preceding symptoms, the use of corticosteroids (n = 25, 57%), and hyperglycemia (n = 28, 82%) were common. Most patients (n = 25, 83%) had received prophylactic or therapeutic antifungals, including voriconazole (n = 17, 39%) and/or an echinocandin (n = 12, 27%).

**Table 1:** Summary of the overall demographic and clinical characteristics of 43 pediatric oncology patients with 44 episodes of mucormycosis, and those of episodes in which Mucorales infection was or was not the primary cause or a contributing factor to mortality.

Variable	All episodes (n = 44)	Mucormycosis-associated deaths (n = 18)	Episodes not resulting in mucormycosis-associated deaths (n = 26)	*P* Value
Median age, yrs (IQR)	12 (8–18)	13 (9–18)	11 (3–18)	0.349
Female (%)	21 (48)	12 (67)	9 (35)	0.065
Underlying diagnosis (%)				1.000
Hematological malignancy	39 (89)	16 (89)	23 (89)	
ALL	19 (43)	8 (44)	11 (42)	
AML/MDS	15 (34)	5 (28)	10 (38)	
Other	5 (11)	3 (17)	2 (8)	
Brain tumor	1 (2)	0 (0)	1 (4)	
Solid tumor	2 (5)	1 (6)	1 (4)	
HSCT for non-malignant disease	2 (5)	1 (6)	1 (4)	
Neutropenia (%)	39 (91)	20 (87)	19 (95)	0.365
HSCT (%)	11 (25)	7 (39)	4 (15)	0.093
Median ANC at onset of infection symptoms (per mm^3^)(IQR)	0 (0–300)	0 (0–570)	0 (0–100)	0.224
Median no. days with ANC<500^a^ (days) (IQR) (n = 36)	23 (4–30)	23 (3–30)	24 (15–28)	0.677
Median no. days with ANC<100^a^ (days) (n = 36)	12 (2–25)	9 (1–25)	13 (4–19)	0.638
Corticosteroid exposure^a^	25 (57)	9 (50)	16 (62)	0.542
Median no. days of corticosteroids (days)(IQR)	15 (5–28)	24 (11–30)	14 (5–26)	0.347
Hyperglycemia^a^ (n = 31)	28 (82)	11 (79)	17 (85)	0.672
Site of infection				0.156
Pulmonary	16 (36)	7 (39)	9 (35)	
Disseminated	14 (32)	6 (33)	8 (31)	
Sinus/orbit/cerebral	5 (11)	4 (22)	1 (4)	
Nasal	1 (2)	0 (0)	1 (4)	
Skin/soft tissue	4 (9)	0 (0)	4 (15)	
Odontogenic	2 (5)	0 (0)	2 (8)	
Gastrointestinal	1 (2)	1 (6)	0 (0)	
Postoperative intracranial	1 (2)	0 (0)	1 (4)	
Etiology (n = 32)^b^				0.909
*Rhizopus* spp.	13 (40)	5/12 (41)	8/20 (40)	
*Mucor* spp.	10 (31)	3/12 (25)	7/20 (35)	
*Cunninghamella* spp.	6 (19)	3/12 (25)	3/20 (16)	
*Absidia* spp.^c^	1 (3)	0/12 (0)	1/20 (5)	
*Lichtheimia corymbifera*	1 (3)	1/12 (8)	0/20 (0)	
*Choaenephora infundibulifera*	1 (3)	0/12 (0)	1/20 (5)	

Abbreviations: ALL, acute lymphoblastic leukemia; AML, acute myelogenous leukemia; ANC, absolute neutrophil count; HSCT, hematopoietic stem cell transplant; IQR, interquartile range; MDS, myelodysplastic syndrome

^a^ Within 30 days prior to onset of symptoms

^b^ One infection was caused by both *Rhizopus* spp. and *Mucor* spp.

^c^ One *Absidia* spp. was not identified to the species level and its nomenclature could not, therefore, be updated to the International Code of Nomenclature adopted by the 18^th^ International Botanical Congress [18].

Symptoms began at a median of 96 days after cancer diagnosis (IQR 27–259 days). Isolated pulmonary (n = 16, 32%) and disseminated infections (n = 14, 32%) were the most frequent forms of infection; rhino-orbital-cerebral infections were relatively uncommon (n = 5, 11%). *Rhizopus* spp. (n = 13, 40%) were the most common pathogens identified, followed by *Mucor* spp. (n = 10, 31%), and *Cunninghamella* spp. (n = 6, 19%). Serious co-infections were present in 3 patients (2 disseminated aspergillosis, 1 disseminated adenovirus).

Empirical or targeted antifungal therapy was administered to all patients, at a median interval of 4 days from symptom onset (IQR 0–6 days) Table 2. Most patients (n = 39, 89%) received amphotericin B (AmB), including 14 (32%) as monotherapy. The deoxycholate (dAmB) formulation was not used after 2000 or as a component of combination therapy. Typical doses of amphotericin deoxycholate and liposomal amphotericin used were 1 mg/kg/day and 3–5 mg/kg/day, respectively. Posaconazole was used as monotherapy in one case, in combination therapy in 22 (50%), and as stepdown therapy in 20 cases (45%). A total of 19 patients (43%) received an echinocandin, generally empirically or in combination with other agents. A total of 23 patients (52%) received additional antifungals that were unlikely to be effective against Mucorales, including flucytosine, itraconazole, and voriconazole. One patient received voriconazole monotherapy. Combination antifungal therapy was prescribed to 25 (57%) of patients, most commonly liposomal AmB (LAmB) + posaconazole (n = 17, 39%), LAmB + an echinocandin (n = 7, 16%), or LAmB + posaconazole + an echinocandin (n = 6, 14%). Among surviving patients prescribed these agents, the median duration of AmB use was 27 days (IQR 7–68), posaconazole 167 days (IQR 131–803), echinocandins 7 days (IQR 6–24), and combination therapy 30 days (IQR 13–46). Trough serum posaconazole concentrations were measured in 16 episodes and were >0.7 μg/ml in 13.

**Table 2:** Summary of the treatment of 43 pediatric oncology patients with 44 episodes of mucormycosis, and those of episodes in which Mucorales infection was or was not considered to be the primary cause or a contributing factor to mortality.

Variable	All episodes (n = 44)	Mucorales-associated deaths (n = 18)	Episodes not resulting in Mucorales-associated deaths (n = 26)	*P* Value
Any antifungal therapy	44 (100)	18 (100)	26 (100)	1.000
AmB (%)	39 (89)	15 (83)	24 (92)	0.386
AmB deoxycholate	12 (27)	8 (44)	4 (15)	**0.045**
Liposomal AmB	28 (64)	8 (44)	20 (77)	0.054
Posaconazole (%)	25 (57)	5 (28)	20 (77)	**0.002**
Echinocandin (%)	19 (43)	7 (39)	12 (46)	0.760
Combination antifungal therapy	25 (57)	6 (33)	19 (73)	**0.014**
Surgery (%)	28 (64)	5 (28)	23 (88)	**<0.001**
Median time to surgery (days) (IQR)	7 (4–13)	10 (7–14)	7 (4–12)	0.306
G-CSF and/or GM-CSF (%)	18 (41)	7 (39)	11 (41)	1.000
Granulocyte transfusion (%)	8 (18)	1 (6)	7 (27)	0.115

Abbreviations: AmB, amphotericin B; G-CSF, granulocyte stimulating factor; GM-CSF, granulocyte-monocyte stimulating factor; IQR, interquartile range

Surgery was performed in 28 episodes (64%) at a median of 7 days after onset of symptoms (IQR 4–13 days); all patients received concurrent antifungal therapy. G-CSF and/or GM-CSF were prescribed in 16 episodes (36%, median 14 doses, range 7–23). Granulocyte transfusions were used in 8 cases (18%, median 3 transfusions per patient). No patient received iron chelation or hyperbaric oxygen therapy.

Median follow up was 167 days (IQR 27–945 days). Overall mortality at 6 weeks, 12 weeks, the end of antifungal therapy, and end of follow up was 29%, 39%, 44%, and 51%, respectively [S2 Table]. Mortality attributable to or contributed to by mucormycosis (mucormycosis-associated mortality) was 27%, 34%, 36%, and 39% at these time points. Survival increased over time, rising to 82% in the period from 2000–2009 from 40–50% in previous decades, although attributable mortality was not significantly associated with decade of diagnosis (OR 0.58, 95% CI 0.33–1.03, p = 0.063).

Antifungal susceptibility testing was obtained for 8 isolates (2 *Rhizopus* spp., 2 *Mucor* spp., and 1 each of *Lichtheimia corymbifera*, *Absidia* sp., *Choanephora infundibulifera* and *Cunninghamella* sp.). Minimum inhibitory concentration ranges for AmB were ≤0.03–4 μg/ml, posaconazole 0.25–1 μg/ml, voriconazole 4–16 μg/ml, isavuconazole >16 μg/ml, micafungin >8 μg/ml, and terbinafine >2μg/ml.

In univariable analyses, treatment with dAmB (OR 4.4, 95% CI 1.07–18.09, p = 0.040) was positively associated with mortality, whereas any use of posaconazole (OR 0.12, 95% CI 0.03–0.46, p = 0.002), combination antifungal therapy (OR 0.18, 95% CI 0.05–0.68, p = 0.011), and adjunctive surgery (OR 0.05, 95% CI 0.01–0.24, p<0.001) were inversely associated with mucormycosis-associated mortality at the end of follow up. Only adjunctive surgery, however, remained independently predictive of survival in multivariable analysis [dAmB (OR 0.51, 95% CI 0.04–7.08, p = 0.620), posaconazole (OR 0.19, 95% CI 0.02–2.29, p = 0.192), combination antifungal therapy (OR 0.47, 95% CI 0.04–5.43, p = 0.542), adjunctive surgery (OR 0.07, 95% CI 0.01–0.36, p = 0.002)]. Of note, establishing a causal relationship between treatment and outcomes of infections in this study is not possible because of its retrospective cohort design.

## Conclusion

The relative rarity of mucormycosis poses challenges to prospective studies of the epidemiology of these infections and to clinical trials to evaluate the efficacy of treatments. Well-performed observational studies, however, may provide useful insights into these conditions.

Together, the current and previous studies of pediatric mucormycosis suggest demographic and clinical characteristics of mycormycosis in children vary according to their underlying medical morbidities and, in children with cancer, across different studies. A male predominance has been reported in most previous case series, although this imbalance was not observed in the current study [4–9]. The mean age of onset of mucormycosis is lower in the general pediatric population than in children with cancer (5–10 vs. 8–14 yrs), reflecting both a relatively large proportion of neonates in the former group and an average age of onset of childhood cancer of 8 years [20]. Rhino-orbital-cerebral infections are reported in 18–28% of pediatric infections overall and gastrointestinal infections in 17–20%, predominantly affecting children with diabetes and neonates, respectively. Skin and soft tissue infection are also relatively common in children overall, representing 15–27% of cases, whereas pulmonary disease is less frequently noted (13–16%). The principal reported sites of infection in children with cancer include lung (5–40%) and nose-orbit-brain (0–93%); skin and gastrointestinal infections are less frequent (0–9% and 0–11% of cases, respectively). Disseminated disease is not defined consistently in the literature but an incidence of 24–32% in all children and 0–50% in children with cancer has been reported. The presence of 2 dental infections in the current series highlights the need to consider mucormycosis in the differential diagnosis of these disorders in children with cancer.

*Rhizopus* spp. is, overall, the most common cause of mucormycosis in children, but the distribution of other species differs across surveys (e.g., *Mucor* spp. are isolated in 0–53% of cases and *Cunninghamella* spp. in 0–28%) [8, 11, 13, 14, 21]. In the current study only 2 infections were caused by *Lichtheimia*/*Absidia* spp., whereas these have been isolated in 4–16% of pediatric infections overall [6, 8, 21]. Interestingly, Pana reported higher rates of *Lichtheimia* spp. infection among children treated in European centers than those treated outside of Europe and suggested that this could be the result of difference in the geographic distribution of fungal species [8]. Species-level differences have practical implications for managing patients, as the Mucorales exhibit different intra- and intergeneric *in vitro* susceptibilities to antifungals–*Rhizopus* spp., for example, are generally less susceptible to amphotericin B than are *Lichtheimia* spp. [22].

The association between mucormycosis and diabetes, particularly diabetic ketoacidosis, is well recognized, but the contribution of poor glycemic control to the risk of mucormycosis in patients with other medical co-morbidities is not clear [1]. Previous reports describe frank diabetes in up to 23% of immunocompromised patients with mucormycosis [9, 23, 24]. Hyperglycemia, related to chemotherapy, high-dose corticosteroids or critical illness, is common in children treated for ALL in particular, and overt hyperglycemia is an independent risk factor for infectious complications and poorer survival in this population [25]. Even in the absence of acidosis, elevated serum glucose may contribute increases in the incidence or severity of mucormycosis by impairing chemotactic responses to hyphae, promoting endothelial cell invasion, and damaging host cells [26, 27]. Further studies to assess outcomes associated with hyperglycemia in this population are warranted, since this may be a frequent and modifiable risk factor for infection.

Most mucormycosis-associated deaths in this series occurred in the setting of unimproved or worsening clinical and radiological disease and within 30 days of the onset of symptoms, but one-third of deaths occurred between 30 and 121 days. Deaths occurring beyond this point, however, were attributed to malignancy or complications of cancer treatment. Mortality in the current case series was comparable to that of previous studies of mucormycosis in children, however it is notable that 7 of 10 patients in the current series who were treated since 2010 survived their infections, including 4 with pulmonary and 2 with disseminated disease [4, 24]. Site of infection, etiologic agent, prior exposure to voriconazole or echinocandins, HCT, and duration of symptoms prior to effective antifungal therapy have been positively or negatively associated with mucormycosis-associated mortality in previous studies, but only adjuvant surgery was significant (protective) in the current series [4, 5, 8, 9, 23]. While the use of adjuvant surgery may be important to infection outcomes, the type and extent of surgery is likely to be of equal importance. It is plausible that the importance of different risk factors for mortality varies across different population, clinical settings, and etiological agents.

Certain biologic characteristics of mucormycosis are likely to contribute to the relatively refractory nature of these infections. Angioinvasion and tissue infarction, in particular, may lead to inadequate tissue concentrations of antimicrobials. Source control may be particularly important in patients with cancer and other persons with impaired innate immune responses; debridement may decrease disease burden by eliminating infected and necrotic tissue. The decision to perform surgery is, however, influenced by the site and extent of infection, patients’ medical co-morbidities, the prognosis of their malignancy, and patient and family preferences. Estimates of the benefits and the risks of tissue debridement, therefore, may be biased because patients with a better overall prognosis are viewed as better candidates for operative therapy. Unacceptably poor outcomes despite intensive antifungal and surgical therapy suggest that novel therapeutic strategies will be necessary to further decrease morbidity and mortality.

This study summarizes one of the largest large single-center experience of mucormycosis in pediatric oncology patients, illustrating that these infections may be heterogeneous, and providing new insights into the epidemiology of these infections in a high-risk population. Strengths include the completeness of data collection, the evaluation of both mucormycosis-attributable and all-cause mortality, the consistent application of inclusion and outcome criteria, the inclusion of only proven cases of infection, and the lengthy and complete follow-up. The long duration of the study, while advantageous in understanding the changing epidemiology and outcomes of mucomycosis in this population, also means that outcomes may be impacted by changes in diagnostic methods and in the management of infections that have occurred over the years. Diagnostic tests, supportive therapy, and even the nomenclature of the mucormycosis have evolved considerably over the past 4 decades. As with previously published retrospective cohort studies of mucormycosis, it is not possible to definitively attribute individual or group outcomes to any specific demographic, clinical, or treatment characteristic. This study was also conducted in a single center and results may not reflect the spectrum of disease in other centers or in different populations, particularly as our data suggest there may be significant variations in causative agents and clinical features across different populations and geographic areas.

## Supporting information

S1 Table Demographic and clinical characteristics of 43 patients with 44 episodes of mucormycosis. (DOCX)

S2 Table Outcome of 44 episodes of Mucorales infection. (DOCX)
