# Supplementary material for: Mucormycosis in children with cancer and hematopoietic cell transplant—A single center cohort study
Source: PLoS One. 2024 Feb 9;19(2):e0297590. doi: 10.1371/journal.pone.0297590 (PMC10857578; doi:10.1371/journal.pone.0297590)
Supplement: S1 Table — (DOCX) [file pone.0297590.s001.docx]

**S1 Table. Demographic and clinical characteristics of 43 patients with 44 episodes of mucormycosis**

| Case | Year | Age (yr)/Sex | Diagnosis | Antifungal exposure ^a^ | Risk factors ^a^ | Site, concurrent infection | Organism | Antifungal therapy (days) | Adjunctive treatment (no.) | Outcomeend of follow up (death, day of illness) |
| --- | --- | --- | --- | --- | --- | --- | --- | --- | --- | --- |
| 1 | 1970 | 8/F | ALL (relapse) | . | N, CS, H ^b^ | Disseminated | *Rhizopus orzyae* | dAmB (2) | Sinus antrostomy | DA (10) |
| 2 | 1972 | 13/M | ALL (previous HD) | . | . | Disseminated | *Rhizopus* spp. | dAmB (15) | None | SC |
| 3 | 1980 | 1/M | ALL (relapse) | . | CS | Skin/soft tissue | Not identified | dAmB (35) | Debridement (3), granulocyte transfusion (2) | SC |
| 4 | 1982 | 18/F | AML/HSCT^c^, GVHD | dAmB | N, CS, H | Gastrointestinal, disseminated *Aspergillosis* | Not identified | dAmB (33) | None | DC (33) |
| 5 | 1983 | 7/F | ALL | . | N, CS, H | Pulmonary | Not identified | dAmB (26) | None | DA (26) |
| 6 | 1985 | 18/M | NHL (relapse) | . | N, CS | Pulmonary | Not identified | dAmB (24) | None | FS (197) |
| 7 | 1989 | 11/F | ALL (relapse) | . | N | Disseminated | *Rhizopus* spp. | dAmB (39), topical dAmB | Ethmoidectomy, maxillary sinusotomy, GM-CSF | DA (43) |
| 8 | 1990 | 18/M | AML | . | N | Pulmonary | Not identified | dAmB (76) | None | DA (121) |
| 9 | 1990 | 8/M | MLL (previous ALL, MDS) | . | N | Pulmonary | *Cunninghamella* spp. | dAmB (14) | None | DA (21) |
| 10 | 1991 | 3/M | AML | . | N | Pulmonary | *Cunninghamella* spp. | dAmB (53) | Partial pulmonary lobectomy | SC |
| 11 | 1993 | 8/F | NB/HSCT (autologous) | . | N | Pulmonary | Not identified | dAmB (23) | G-CSF | DA (28) |
| 12 | 1999 | 1/M | HSCT-SCID, GVHD | . | N, CS | Pulmonary, pulmonary *Aspergillosis* | *Cunninghamella* spp. | plAmB (27) | Pulmonary lobectomy, granulocyte transfusion (14) | SC |
| 13 | 2000 | 18/M | tAML (previous ALL) | . | N | Sinus orbital | Not identified | dAmB (25), plAmB (9), LAmB (3) | Bilateral ethmoidectomy, maxillary antrostomy, GM-CSF, G-CSF | DC (52) |
| 14 | 2000 | 8/F | AML | . | N, H | Pulmonary | *Rhizopus* spp*.* | LAmB (78) | Pulmonary lobectomy, thoracostomy | DC (109) |
| 15 | 2001 | 9/F | ALL | . | N, CS, H | Disseminated | *Mucor* spp*.* | LAmB (90) + CASP (37) | Debridement (3), G-CSF | SC |
| 16 | 2002 | 10/F | ALL (relapse) | VORI, LAmB | N, CS, H | Rhinocerebral | Not identified | VORI (21) | None | DC (21) |
| 17 | 2003 | 21/M | ALL (relapse) | Casp | N, CS, H | Disseminated | *Mucor* spp*.* | LAmB + CASP (6),  LAmB + POSA (334),  POSA (577) | Partial pulmonary lobectomy, G-CSF, granulocyte transfusion (2) | SC |
| 18 | 2004 | 9/F | ALL (relapse) | VORI, CASP | N, CS, H | Odontongenic | *Rhizopus* spp*.* | LAmB + POSA + CASP (8),  LAmB + POSA (35) | Dental extraction, G-CSF, granulocyte transfusion (3) | DN (52) |
| 19 | 2004 | 9/F | MDS | VORI | . | Disseminated | *Cunninghamella* spp*.* | LAmB (20), CASP (2),  POSA + CASP (9),  POSA (14) | G-CSF | DC (46) |
| 20 | 2005 | 19/M | AML | VORI | N, H | Disseminated | *Mucor* spp*.* | CASP (4),  LAmB (6), LAmB + POSA (40),  POSA (196) | Partial pulmonary lobectomy, G-CSF, granulocyte transfusion (11) | SC |
| 21 | 2005 | 4/M | NB/HSCT (autologous) | . | N | Endontogenic | *Rhizopus* spp*.* | LAmB (5),  LAmB + POSA (12),  POSA (506) | Dental extraction, debridement, G-CSF | SC |
| 22 | 2006 | 18/F | HSCT - HbSS | VORI | CS, H | Disseminated | *Cunninghamella* spp*.* | LAmB + MICA (3),  LAmB + POSA + MICA (8) | G-CSF | DA (21) |
| 23 | 2007 | 1/M | AML | VORI | N, CS, H | Skin/soft tissue | *Rhizopus* spp*.* | POSA + MICA (4),  LAmB (7),  POSA (160) | None | SC |
| 24 | 2008 | 15/F | ASTRO | . | N, CS | Postoperative intracranial | *Rhizopus* spp*.* | POSA + MICA (45),  POSA (573) | Debridement, GM-CSF | SC |
| 25 | 2008 | 15/M | ALL (relapse)/HSCT, GVHD | MICA | N, CS, H | Disseminated | *Mucor* spp*.* | LAmB + MICA (2) | None | DA (2) |
| 26 | 2008 | 9/F | MLL (relapse) | MICA | N, CS, H | Pulmonary | *Rhizopus* spp*.* | MICA (8),  LAmB + POSA (5) | Debridement (3), GM-CSF | DA (13) |
| 27 | 2009 | 17/F | ALL/HSCT, GVHD | MICA | N, CS, H | Sino-orbital | *Mucor* spp*.* | MICA (4) | None | DA (5) |
| 28 | 2010 | 1/M | ALL | . | N, CS, H | Disseminated | Not identified | MICA (7),  LAmB + MICA (4),  LAmB + POSA + MICA (229),  LAmB + POSA (70),  POSA (504) | Partial pulmonary lobectomy, partial nephrectomy | SC |
| 29 | 2010 | 18/F | MLL (relapse)/HSCT | VORI | N | Rhinosinusitis, disseminated adenovirus | *Mucor* spp*.* | MICA (2),  LAmB + MICA (35) | Granulocyte transfusion (4) | DC (41) |
| 30 | 2010 | 12/M | ALL | VORI | N | Pulmonary | Not identified | MICA (7),  POSA (796) | Partial pulmonary lobectomy, debridement | SC |
| 31 | 2011 | 18/M | AML | VORI | N | Pulmonary | *Rhizopus microsporus var. chinensis* | LAmB + POSA (7),  LAmB + POSA + MICA (16), POSA (76) | Partial pulmonary lobectomy, G-CSF, granulocyte transfusion (2) | SC |
| 32 | 2011 | 7/F | ALL | MICA | N, CS, H | Disseminated | *Absidia* spp*.* | LAmB + POSA + MICA (7),  LAmB + POSA (61),  POSA (960) | Partial pulmonary lobectomy | SC |
| 33 | 2012 | 14/F | AML | VORI | N, H | Pulmonary | *Mucor* spp*.* | LAmB + POSA + MICA (24), POSA (112) | Bilateral pulmonary lobectomy, G-CSF | SC |
| 34 | 2013 | 15/M | ALL | . | N, CS, H | Pulmonary | *Cunninghamella* spp*.* | LAmB + MICA (6),  LAmB + POSA (39),  POSA (112) | Partial pulmonary lobectomy | SC |
| 35 | 2013 | 10/M | ALL (relapse) | MICA, VORI | N, CS, H | Pulmonary | *Rhizopus* spp*.* | LAmB (3), LAmB + POSA (34),  POSA (2) | Partial pulmonary lobectomy, G-CSF | DN (44) |
| 36 | 2014 | 18/F | AML | VORI | N, CS, H | Pulmonary | Not identified | LAmB (4), LAmB + POSA (24),  POSA (44) | Partial pulmonary lobectomy | SC |
| 37 | 2014 | 5/M | AML/HSCT | MICA | N, CS, H | Rhinosinusitis | Not identified | LAmB + POSA (16),  POSA (399) | Debridement | DN (417) |
| 38 | 2015 | 14/M | ALL/HSCT, GVHD | VORI | CS, H | Disseminated | *Lichtheimia corymbifera* | POSA + MICA (24),  POSA (51), LAmB + POSA (107) | None | DA (107) |
| 39 | 2015 | 15/F | AML | MICA, VORI | N, H | Disseminated | *.* | LAmB (2),  POSA (167) | Debridement | SC |
| 40 | 2016 | 17/M | ALL | MICA, VORI | N, H | Pulmonary | *Rhizopus* spp*.* | POSA (7) | GM-CSF, G-CSF | DA (7) |
| 41 | 2016 | 3/F | ALL | MICA | N, CS, H | Skin/soft tissue | *Mucor* spp*.* | POSA (27), LAmB + POSA (19),  POSA (85) | Debridement (3), GM-CSF | SC |
| 42 | 2016 | 1/M | MLL (relapse)/HSCT | VORI | N, CS, H | Rhinosinusitis | *Mucor* spp*.* | LAmB + POSA (6),  LAmB (16) | Debridement (3)  G-CSF | DN (22) |
| 43 | 2019 | 21/F | AML | VORI | N, H | Skin/soft tissue/bone | *Rhizopus* spp. + *Mucor* spp. + *Fusarium* spp. | LAmB (5), LAmB + POSA (49),  POSA (49) | Debridement (3), partial amputation, G-CSF, granulocyte transfusion (3) | SC |
| 44 | 2019 | 18/M | AML | MICA | N, H | Disseminated | *Choanephora infundibulifera* + *Curvularia* spp. | LAmB + POSA (36),  LAmB (55),  POSA (45) | Debridement (5) | DN (137) |

Abbreviations: 5-FC, 5-flucytosine, ALL, acute lymphoblastic leukemia, AML, acute myelogenous leukemia, ASTRO, astrocytoma, CASP, caspofungin, CS, corticosteroid, dAmB, amphotericin B deoxycholate, DA, death, attributable to Mucorales infection, DC, death, Mucorales infection contributing, DN, death, not related to Mucorales infection, FLU, fluconazole, G-CSF, granulocyte stimulating factor, FS, failure - stable response, GM-CSF, granulocyte-monocyte stimulating factor, GVHD, graft versus host disease, HSCT, hematopoietic stem cell transplantation, H, hyperglycemia, ITRA, itraconazole, LAmB, liposomal amphotericin B, MDS, myelodysplastic syndrome, MICA, micafungin, MLL, mixed lineage acute leukemia, N, neutropenia, NB, neuroblastoma, NHL, non-Hodgkin lymphoma, plAmB, amphotericin B phospholipid complex, POSA, posaconazole, PR, partial clinical and/or radiological response to therapy, SC, success - complete response, SCID, severe combined immunodeficiency, tAML, treatment-associated AML, VORI, voriconazole

^a^ Within 30 days prior to onset of symptoms

^b^ Hyperglycemia defined as any serum glucose concentration exceeding the normal reference range for age

^c^ Allogeneic HSCT unless otherwise noted
