# Supplementary material for: Mucormycosis in children with cancer and hematopoietic cell transplant—A single center cohort study
Source: PLoS One. 2024 Feb 9;19(2):e0297590. doi: 10.1371/journal.pone.0297590 (PMC10857578; doi:10.1371/journal.pone.0297590)
Supplement: S2 Table — (DOCX) [file pone.0297590.s002.docx]

**S2 Table. Outcome of 44 episodes of Mucorales infection.**

| Outcome | No. (%) |
| --- | --- |
| Infection status at 6 weeks |  |
| Success, complete response | 7 (16) |
| Success, partial response | 14 (31) |
| Failure, stable response | 4 (9) |
| Failure, progression of disease | 7 (16) |
| Death, all-cause | 12 (27) |
| Death, Mucorales-associated | 12 (27) |
| Infection status at 12 weeks |  |
| Success, complete response | 10 (23) |
| Success, partial response | 11 (25) |
| Failure, stable response | 4 (9) |
| Failure, progression of disease | 2 (5) |
| Death, all-cause | 17 (39) |
| Death, Mucorales-associated | 10 (23) |
| Infection status at end of therapy |  |
| Success, complete response | 18 (41) |
| Success, partial response | 4 (9) |
| Failure, stable response | 2 (5) |
| Failure, progression of disease | 1 (2) |
| Death, all-cause | 19 (43) |
| Death, Mucorales-associated | 16 (36) |
| Infection status at end of follow up |  |
| Success, complete response | 20 (45) |
| Success, partial response | 1 (2) |
| Failure, stable response | 1 (2) |
| Failure, progression of disease | 0 (0) |
| Death, all-cause | 22 (50) |
| Death, Mucorales-associated | 17 (39) |
